# Performance of the ADAPT-Treated CardioCel® Scaffold in Pediatric Patients With Congenital Cardiac Anomalies: Medium to Long-Term Outcomes

**DOI:** 10.3389/fped.2020.00198

**Published:** 2020-04-24

**Authors:** William Neethling, Alethea Rea, Guenther Forster, Kiran Bhirangi

**Affiliations:** ^1^School of Surgery, University of Western Australia, Perth, WA, Australia; ^2^Department of Cardiothoracic Surgery, University of Free State, Bloemfontein, South Africa; ^3^Centre for Applied Statistics, University of Western Australia, and Mathematics and Statistics, Murdoch University, Perth, WA, Australia; ^4^ADMEDUS Pty LTD, Bangkok, Thailand; ^5^ADMEDUS Pty LTD, Minneapolis, MN, United States

**Keywords:** congenital, cardiac defects, bovine, pericardium, calcification

## Abstract

**Background:** A Phase II Clinical Trial reviewed the performance (morbidity and calcification) of the tissue-engineered ADAPT® bovine pericardial scaffold (CardioCel®) in pediatric patients (*n* = 30) with congenital cardiac defects. In that study, CardioCel® demonstrated no graft-related morbidity and mortality in 25 patients, over 12 months. Five patients died due to non-graft-related events. Echocardiography revealed hemodynamically stable repairs with no calcification of the scaffold. Magnetic resonance imaging (MRI) at 12 months in 10 patients confirmed the absence of calcification. These patients were followed up for further up to 10 years. We present the results of this retrospective review of these patients that were followed for further medium to long-term (median 7.2 years, 25%: 3.6 years 75%: 9.25 years) postoperatively in these patients.

**Methods:** Between April 2008 and September 2009, CardioCel® was implanted in 30 patients with congenital cardiac defects. Efficacy measures included graft-related mortality, morbidity and haemodynamic abnormalities. Calcification was assessed by standard 2D-M mode echocardiography and MRI at 12 months. Medium to long-term assessment included routine clinical assessments and echocardiography.

**Results:** Median age at surgery was 18 months (27 days−13 years). Twenty-five patients (142 patient years) were followed for up to 10 years. The 10-year survival rate is estimated as 86.9% (95% CI 71.4–100.0%) over the entire follow-up period. One patient was lost to follow-up. No graft-related mortality was encountered up to a median follow-up of 7.2 years. Two patients died (pacemaker complications >5 years and arrhythmia >7 years postoperatively). No graft failure, thromboembolic events, infections or device-related reinterventions were recorded. Non-significant residual leaks occurred in 3 patients. Echocardiography demonstrated the absence of calcification in all implants.

**Conclusion:** The tissue-engineered ADAPT® bovine pericardial scaffold demonstrated excellent medium to long-term performance (up to 10 years) when used as a scaffold for repair of congenital cardiac defects in children. Durability, acellularity, biostability and non-calcifying potential of CardioCel® makes it a very attractive tissue for congenital cardiac repair procedures.

## Introduction

Since 1977, a variety of bioprosthetic substitutes have been used for pediatric cardiac repairs such as bovine and porcine pericardium fixed with glutaraldehyde, synthetics such as Dacron®, cryopreserved homografts and autologous pericardium, fresh or treated with glutaraldehyde (1, 2).

Some of these materials failed due to intimal hyperplasia, calcification, shrinkage with less pliability, resulting in a compliance mismatch with surrounding native tissue (3).

Calcification of Dacron and xenogeneic substitutes fix with glutaraldehyde after implantation is well-known while fresh pericardium tends to dilate with resulting aneurysm formation in high pressured areas (3, 4) and shrinkage of extended valve leaflet augmentation (5).

Bovine pericardium fixed with glutaraldehyde tends to degenerate when these implants become rigid due to calcium deposits and develop changes in the structural integrity (shape and size) after implantation which eventually causes failure of these grafts. Toxicity caused by residual glutaraldehyde (6) and induced immune responses of the recipient (7) tend to be the main causes responsible for degeneration and calcification of these implants.

While the atrioventricular septal defect (ASD) and ventricular septal defect (VSD) have a low incidence of complications after septal closure, more significant adverse events such as thrombus formation on synthetic matrices (8), aneurysms occurring in biological substitutes (9) and dehiscence of calcified patches have been well published (10).

The present study was performed to assess the performance of the tissue-engineered ADAPT®-treated bovine pericardial scaffold over a mid-term follow up period extending to 10 years in a series of congenital cardiac repairs.

## Patients and Methods

In the prospective, non-randomized Phase II Clinical study, a range of congenital cardiac anomalies in 30 pediatric patients were repaired with ADAPT®-treated tissue-engineered bovine pericardial scaffold (Cardiocel®, ADMEDUS Pty Ltd, Perth Western Australia) from April 2008 to September 2009 at the Universitas Hospital, Faculty of Health Science, University of Free State in Bloemfontein, South Africa. The study was registered (clinical registration number ETOVS NR 27/7) and approved by the Ethics Committee of the Faculty of Health Science, University of Free State, Bloemfontein, South Africa. Formal consent was obtained from all patients next of kin or legal representative. Patients were eligible for inclusion if ADAPT®-treated tissue-engineered Cardiocel® was used as a bioprosthetic substitute during repair of congenital heart defects using cardiopulmonary bypass. A range of congenital cardiac repairs included ASD, VSD, atrioventricular septal defect (AVSD), aortic root enlargement and right ventricular outflow tract (RVOT) reconstruction. All repair procedures were performed by the same two surgeons. Patients were followed for 12 months during the initial study period and these results been previously published (11).

The ADAPT®-treated tissue-engineered Cardiocel® scaffold is manufactured from bovine pericardium which is certified as BSE (bovine spongiform encephalopathy) free. The manufacturing processes of Cardiocel® consists of several tissue engineering steps which remove phospholipids, cells and cell remnants within the scaffold, nucleic acids (DNA, RNA) and α-Gal epitopes. An ultra-low concentration of chemically engineered, monomeric glutaraldehyde is used to cross-link the scaffold to minimize the potential cytotoxicity levels of glutaraldehyde. An additional anti-calcification process ADAPT® process (ADMEDUS Pty Ltd proprietary) is applied to further reduce the glutaraldehyde cytotoxicity to zero levels. Sterilization of the Cardiocel® scaffold is performed with a non-glutaraldehyde solution and stored in the same solution which provides additional anti-calcification properties (11).

This study is a retrospective review of the mid-term follow up those patients who underwent surgical repair of a congenital heart defect, using tissue-engineered CardioCel® bovine pericardium up to 10 years of follow up. The medical records of each patient were reviewed, and the following patient and scaffold-related data collected: gender, age at operation, primary cardiac diagnosis, number, type and location of patches, and echocardiographic assessment reports.

All patients (24/30) received routine echocardiographic assessments during each follow-up visit which were reported by two experienced pediatric cardiologists. Calcification was assessed by standard 2D-M mode echocardiography (HP3000, Hewlett Packard, JHB, South Africa) by the most experienced sonographer (16 years of experience in pediatric echocardiography) who was blinded from the clinical information. A numeric scale of 0 (no visible calcification), 1 (mild, focal calcification), or 2 (moderate to severe dispersed calcification) based on increased echo density differences between the site of implantation and surrounding tissue was used to identify the level of calcification.

The primary endpoints included death of the patient, failure of the tissue scaffold resulting in reoperation and/or any form of scaffold-related reintervention.

### Statistical Analysis

Patient demographic and baseline characteristics were summarized as frequencies and percentages for categorical variables and continuous variables were expressed as median (range). As the cohort is small, statistical analysis was limited to a Kaplan-Meier plot containing the survival distribution of 24 patients and a Cox regression performed on the time to last follow up or survival. The 10-year survival rate is presented with a 95% confidence interval to capture the uncertainty in the estimate. R and the package survival were used for analysis.

## Results

A total of 34 CardioCel® devices were implanted in 30 patients. The baseline patient characteristics and distribution of cardiac anomalies are summarized in Table 1. The median age at the time of surgery was 18 months (range, 27 days to 13 years, 3 months).

**Table 1:** Patient demographics and baseline characteristics.

All implants, n (%)	30 (100)
Median age, months (range)	18 (27 days−13 years, 3 months)
Male, n (%)	17 (57)
Etiology, n (%)	
ASD	1 (3)
VSD	14 (47)
AVSD	3 (10)
RVOT	2 (7)
ASD and VSD	1 (3)
VSD and RVOT	4 (13)
ASD, VSD and RVOT	1 (3)
Vascular patch (aorta)	2 (7)
VSD and coarctation	2 (7)
NYHA functional class, n (%)	
I	20 (67)
II	7 (23)
III	2 (7)
IV	1 (3)

*ASD, Atrial septal defect; VSD, Ventricular septal defect; AVSD, Atrioventricular septal defect; RVOT, Right ventricular outflow tract*.

Cardiac diagnoses included VSD (*n* = 14, 47%), VSD + RVOT (*n* = 4, 13%), AVSD (*n* = 3, 10%), trans-position of the great arteries (TGA) (*n* = 2, 7%), double outflow right ventricle (DORV) (*n* = 2, 7%), truncus arteriosus (TA) (*n* = 2, 7%), aortic arch (Ao) (hypoplastic arch + coarctation *n* = 1, 3%; coarctation *n* = 1, 3%) and ASD (*n* = 1, 3%). Concomitant procedures included mitral valve plasty in one patient (3%).

Follow-up was available on 24 patients (80%). The median follow-up was 7.2 years (range 1.3–10.6). Seven patients (28%) had follow up periods between 1 and 5 years and 17 (68%) patients were followed up for between 5 and 10 years (Table 2). One patient was lost to follow-up after the initial clinical trial period of 12 months. The geographical relocation and difficulties with traveling from remote areas were cited as reasons for the lack of follow-up, a known difficulty for clinical trials in South Africa.

**Table 2:** Patient follow-up 1–10 years.

**Diagnosis**	**Application**	**Follow-up (yrs)**
**Follow-Up 1–5 years (*****n*** **=** **7)**		
ASD + VSD	ASD + VSD	3.4
ASD	ASD	1.4
VSD	VSD	3.5
VSD	VSD	3.7
VSD	VSD	1.8
VSD	VSD	2.3
VSD	VSD	0.8
**Follow-Up 5–10 years (*****n*** **=** **17)**		
Tetralogy of fallot	VSD + RVOT Trans-annular Patch	10.4
DORV	Trans-annular Patch	7.0
Truncus art	VSD	9.4
TGA	VSD	9.3
TGA	VSD + Transannular Patch	5.2
Ao Coarctation + VSD	Aortic patch + VSD	9.4
Ao Coarctation	Aortic patch	8.5
AVSD + Mitral valve stenosis	AVSD + Mitral valve repair	9.5
AVSD	AVSD	9.1
VSD + Pulm stenosis	VSD + Transannular Patch	7.4
VSD	VSD	8.4
VSD	VSD	6.3
VSD	VSD	6.4
VSD	VSD	9.8
VSD + PDA	VSD	9.2
VSD	VSD	5.2
VSD	VSD	7.7

*ASD, Atrial septal defect; VSD, Ventricular septal defect; AVSD, Atrioventricular septal defect; DORV, Double outlet right ventricle; TGA, Transposition of the great arteries*.

Stable non-haemodynamically significant small residual VSDs between 2.0 and 3.1 mm were detected during echocardiography assessments in 3 (10%) patients during follow up.

None of the transannular patches in the LVOT (*n* = 2) or RVOT (*n* = 4) demonstrated flow obstruction during echocardiographic assessment.

**Figure 1:**
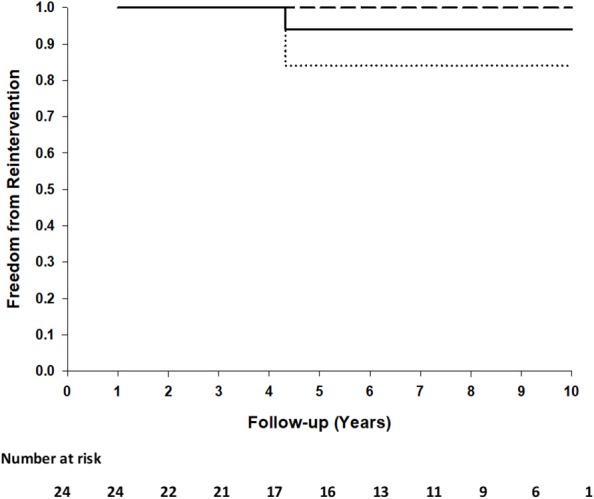
Kaplan-Meier plot of freedom from reintervention of 24 patients. Ninety five percentage of confidence interval (dashed lines) at 10 years.

No surface thickening, structural leaks or calcification in any of these implants were detected. There were no thromboembolic events, no device-related re-operations, no device replacements or no graft failures in any patients. One patient (AVSD + mitral valve plasty) had a mitral valve replacement 4 years after the initial procedure. Examination of the AVSD repair area during the mitral valve procedure revealed an intact repair without any surface thickening, indications of dehiscence or obstruction. Freedom from reintervention at 5 and 10 years is 0.94 (95%CI: 0.84–1.00) (Figure 1).

**Figure 2:**
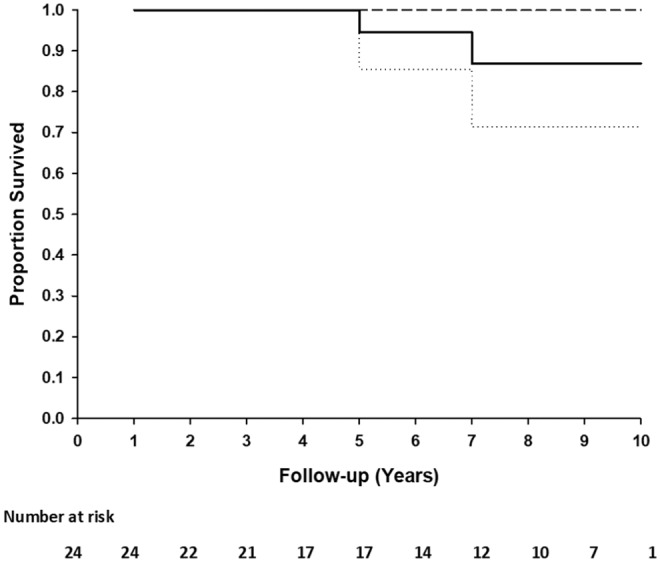
Kaplan-Meier plot of the survival distribution of 24 patients. Ninety five percentage of confidence interval (dashed lines) at 10 years.

Two patients died due to comorbid non-graft-related events. One patient (TGA + VSD) died at 5.2 years postoperatively due to acute pacemaker failure and one patient (DORV) died 7 years postoperatively due to uncontrolled arrythmia. Freedom from death at 5 and 10 years is 1.00 (95%CI: 1.00–1.00) and 0.71 (95%CI: 6.99–1.00), respectively (Figure 2). No patient underwent reoperation for a CardioCel related problem. Calcification of the implant was not detected on echocardiography in any patient.

## Discussion

In this single-center, retrospective study, we have demonstrated medium to long term durability, efficacy and the absence of calcification of the tissue-engineered ADAPT® bovine pericardial scaffold (CardioCel®) when used to repair a variety of congenital heart defects in pediatric patients followed for up to 10 years.

The use of several synthetic and bioprosthetic materials (homografts, pericardial xenografts and porcine intestinal sub-mucosa) for cardiovascular applications and the repair of congenital heart deformities have been well-documented (12). To our knowledge, none of these materials have specifically been followed over extended implantation time. The ideal characteristics of these bioprosthetic substitutes should include easy handling, efficient pliability to allow optimal manipulation without being too flexible that would allow folding on itself, stable after implantation, resistant to calcification and non-sensitizing to the immune system. At present, none of the available patch materials have all these characteristics (13). Fresh autologous pericardium tends to be an attractive option due to advantages such as immediate availability, low-cost, the absence of donor-derived pathogens and non-immunogenicity (4). It provides satisfactory long-term results when used for repair of simple congenital defects and for aortic valve repair when rapidly fixed with glutaraldehyde (5, 14). However, when used without glutaraldehyde fixation, it shrinks, develops fibrosis and tends to stretch when exposed to high cardiovascular pressure, with reports of aneurysm formation at the site of the repair (13). The main advantage of glutaraldehyde fixation is that it improves the biostability of autologous and xenogeneic pericardium through collagen crosslinking, but with the potential consequences of residual toxic aldehydes which could result in late calcification (5, 15). However, structural degeneration and dystrophic calcification remain limiting factors in terms of the longevity and clinical effectiveness of these bioprosthetic substitutes (11, 12, 16). The inability of these materials to grow with the recipient tissue, loss of tensile strength over extended implantation time and inability to remodel or regenerate further reduce the potential for long-term performance. Bioprosthetic substitutes are sometimes associated with a risk of the infection and the formation of aneurysms, in addition to the cytotoxic effects of various chemical cross-linking agents (17).

To date, most of the retrospective studies with bovine pericardial patches in congenital cardiac surgery are limited to case reports or short-term results (11, 13). Several case reports of complex congenital cardiac repairs with CardioCel® demonstrated favorable outcomes in terms of handling characteristics, durability and clinical efficiency (18–20).

In addition, short to medium term retrospective studies with Cardiocel have also demonstrated positive outcomes in terms of performance, no calcification and efficacy (21–24). A less favorable outcome (freedom from reoperation 57% at 36 months) was reported in a single center study of CardioCel® used in aortic valve reconstruction in young children (25). On the other hand, a more promising outcome was reported by Bell et al. (23) with freedom from reintervention in 135 patients (140 procedures and 195 implants) at 36 months of 94% (CI 89–97) without any evidence of calcification after echocardiographic and radiological assessment in any of these patients. Similar results were reported in a multi-center study (Brisbane, Australia; and Leicester and Bristol, United Kingdom) with an overall freedom from reintervention at 3 and 5 years after implantation of 96% (95% confidence interval, 93–98%) (24).

One of the most important outcomes with CardioCel® is clinical evidence of early remodeling (scattered capillary vessels with new collagen fibers and formation of neointima) which was demonstrated in explanted samples with implantation time ranging from 67 to 502 days (21).

In the present study, the medium to long-term performance of CardioCel®, in a variety of congenital cardiac repairs have retrospectively been assessed between 1 and 10 years. The most outstanding findings of this study were the absence of graft-related adverse events and calcification.

## Limitations

This study has all the limitations of a small retrospective study. It is a single-center study without a control group. The main limitation of the study was a small number of patients and follow-up was incomplete in some patients. Another limiting factor was the heterogenous patient groups based on the different areas of application. The use of echocardiography to detect calcification was also be regarded as a limitation in view of the scoring system which assesses semi-quantitatively the presence of hyperechoic zones or changes in echodensity as opposed to a quantitative measurement with a computed tomography (CT) scan.

## Conclusion

In summary, this study shows that in these pediatric patients with a range of congenital cardiac anomalies, followed for 1–10 years, the tissue engineered ADAPT® bovine pericardial scaffold (CardioCel®) demonstrated good biocompatibility and durability. No graft-related calcification was observed, and there was no graft-related morbidity or mortality reported.

## Data Availability Statement

The raw data supporting the conclusions of this article will be made available by the authors, without undue reservation, to any qualified researcher.

## Ethics Statement

Signed informed consent was obtained from all patients next of kin or legal representatives. This study was approved by the Ethics Committee of the Faculty of Health Science, University of Free State, Bloemfontein, South Africa (Registration number ETOVS NR 27/7).

## Author Contributions

WN: principle investigator, design and layout of manuscript, interpretation of data, and writing of manuscript. AR: consultant statistician responsible for statistical analyses. GF: assistance with writing and revision of the manuscript and final approval. KB: assistance with writing of the manuscript, revising the manuscript, and final approval.

## Conflict of Interest

The authors declare that the initial prospective non-randomized clinical trial was funded by Celxcel Pty Ltd [previous owner of the ADAPT Technology – (11)]. ADMEDUS Pty Ltd. The funder was not involved in the study design, analysis, interpretation of data, the writing of this article or the decision to submit it for publication. The funder paid for MRI procedures, donated the Cardiocel devices for the study and paid for travel expenses to collect data at the institution where the study was conducted. The medium to long-term follow-up study was non-funded and executed as an academic research program which involved University of Western Australia, University of Free State with technical support from Admedus Pty Ltd. WN is Professor at School of Surgery, University of Western Australia and advisor to Admedus as VP Cardiovascular Technologies of ADMEDUS Pty Ltd. KB is consulting Chief Medical Officer for ADMEDUS Pty Ltd. GF is consulting Medical Director of ADMEDUS Pty Ltd, Asia Pacific Region. The remaining author declares that the research was conducted in the absence of any commercial or financial relationships that could be construed as a potential conflict of interest.
